# Early detection and intervention in dermatomyositis: current evidence and clinical implications

**DOI:** 10.1007/s10067-026-07971-w

**Published:** 2026-02-10

**Authors:** Kenneth Treasure, Rehet Chugh, Hana Abbas, Arpita Patel

**Affiliations:** 1 Burrell College of Osteopathic Medicine, 3501 Arrowhead Dr, Las Cruces, NM 88001 USA; 2 Meharry Medical College School of Medicine, Nashville, TN USA; 3 Kansas City University College of Osteopathic Medicine, Kansas City, MO USA

**Keywords:** Calcinosis cutis, Dermatomyositis, Early diagnosis, Immunotherapy, Multidisciplinary care, Muscle atrophy

## Abstract

Dermatomyositis is a rare autoimmune disease characterized by muscle weakness and distinctive skin rashes. Patients typically present with symmetric proximal muscle weakness alongside extramuscular manifestations, including interstitial lung disease and skin infections. While the exact cause remains unknown, researchers believe genetics, infections, and underlying autoimmune processes contribute to its development. The disease likely involves inappropriate complement protein activation targeting the perimysium, which triggers blood vessel inflammation and destruction around muscle fascicles. This process leads to ischemia and infarction of muscle tissue, ultimately causing muscle inflammation and atrophy. This paper analyzes existing data to determine whether early diagnosis and treatment of dermatomyositis reduce musculoskeletal complications. Several factors contribute to diagnostic delays: dermatomyositis occurs less frequently than other myopathies, early symptoms remain non-specific, and accurate diagnosis requires combining clinical, serologic, and muscle biopsy findings. Current evidence demonstrates that early detection and treatment of autoimmune diseases like dermatomyositis result in higher remission rates and reduced disease activity.

## Introduction

*Dermatomyositis (DM)* can be characterized as a *rare idiopathic inflammatory myopathy* that presents with *cutaneous rashes* and *progressive muscular weakness* [1]. *Delayed diagnosis* has been associated with *irreversible muscle damage*, *functional impairment*, and a *diminished quality of life*. Dermatomyositis is a condition where diagnostic and therapeutic delays significantly affect prognosis. *Chronic systemic inflammation* can lead to the development of *interstitial lung* and *cardiovascular disease*, as well as create an optimal environment for *malignancies* to grow.

For an accurate diagnosis, clinicians must be able to recognize *pathognomonic skin findings*, such as *Gottron’s papules*, violaceous papules that tend to appear on the knuckles, knees, and elbows [2]. Additionally, the presence of *heliotrope rash*, which can be characterized as *bilateral lilac discoloration of the eyelids* with *swelling around the eye*, is highly characteristic of DM [3]. Research shows that a *heliotrope rash* can be used as an *early indicator* for DM, differentiate DM from other *inflammatory myopathies*, as well as *monitor disease activity* based on rash appearance [4]. It is also recommended that patients undergo a thorough assessment of *muscle strength and function* because abnormalities in these areas can indicate *muscle damage* [4]. Lastly, accurate and timely diagnosis of dermatomyositis can be difficult for clinicians because of how *rare* the disease is, with many clinicians seeing *few cases* throughout their entire career.

In order for clinicians to confidently differentiate dermatomyositis from other myopathies, they should utilize a *comprehensive diagnostic approach* involving *serological analysis*, *coordination across multiple specialties*, and *tissue biopsies*. To reliably differentiate dermatomyositis from other myopathies, clinicians should employ a *comprehensive diagnostic approach that incorporates serologic testing*,* multidisciplinary evaluation*, and *tissue biopsy when indicated.* Multiple studies highlight that the foundation of *early intervention* involves the initiation of *immunosuppressive therapy*, most often *glucocorticoids*, which can be used to suppress the *inflammatory cascade*, preventing further muscle damage [5]. However, early diagnosis may not be possible for all patients due to *systemic barriers*.

An individual’s *socioeconomic status* plays a significant role in their ability to access care. Treatment for dermatomyositis includes *steroid-sparing medications* such as *methotrexate* or *azathioprine*, which may also be part of the treatment plan for individuals who are at increased risk of *glucocorticoid-related adverse effects* [6]. The goal of this literature review is to assess the role of *early detection* in improving *clinical* and *musculoskeletal outcomes*. This paper aims to explore several key questions: *How does early diagnosis influence disease progression and physical function? What barriers hinder timely identification? And what strategies can enhance early detection and expedite treatment initiation?*

## Pathophysiology

### Immune-mediated mechanism

Dermatomyositis is considered a *systemic autoimmune disorder*, which is defined by the adaptive immune responses, involving both humoral and cellular responses. Dermatomyositis is a systemic autoimmune disorder characterized by adaptive immune activation involving both humoral and cellular responses. A distinctive feature of DM is *capillary-targeted injury* caused by complement activation and characterized by both cutaneous and muscular microvascular damage [7]. The *C5b-9 membrane attack complex (MAC)* forms and deposits onto the endothelium of capillaries, causing microangiopathy, capillary necrosis, and perifascicular ischemia. Eventually, muscle fibers at the edges of fascicles begin to atrophy, resulting in the hallmark perifascicular atrophy seen in dermatomyositis histopathology, which is why *perifascicular atrophy* is seen in biopsies for DM [8]. The perifascicular atrophy is a direct result of hypoperfusion and ischemic injury to muscle fibers due to the microangiopathy, which also explains why DM is categorized as a *complement-mediated vasculopathy* rather than a T-cell-mediated muscle disease.

However, the immune dysfunction goes beyond that of complement dysfunction because it includes the adaptive immune response, with B cells and CD4 + T cells entering perimysial and perivascular spaces. These cells are rich with *plasmacytoid dendritic cells*, which are strong inducers of *type I interferon (IFN-α/β) signaling*. Type I interferons are cytokines that are consistently elevated in DM, proven by the presence of IFN-induced genes such as MxA in biopsies, sustaining inflammation and amplifying immune activation [9]. B cells also produce autoantibodies that further activate the complement pathway, amplifying the immune response and contributing to autoimmunity, which is evident by the presence of plasmablasts.

### Myositis-specific autoantibodies (MSAs)

Many *myositis-specific autoantibodies (MSAs)* are now being utilized as not only diagnostic tools but also as indicators for different clinical immunopathologic subtypes. *Anti-Mi-2 antibodies* are associated with the classical cutaneous features of DM and are linked to favorable outcomes [10]. On the other hand, *anti-MDA5 antibodies* are predictive of clinically amyopathic dermatomyositis (CADM), cutaneous involvement without muscle atrophy, and they also predict an increased risk of rapidly progressive interstitial lung disease (RP-ILD). *Anti-TIF1-γ (p155/140) autoantibodies* are linked to paraneoplastic syndromes, which extensively amplify cutaneous involvement and are commonly seen in DM among adults [11]. These antibodies can play a role in both initiating the disease and amplifying immune responses by disrupting normal cellular processes while enhancing Type I interferon signaling. Specific dermatomyositis markers can be derived from recognizing myositis-specific autoantibodies and myositis-associated autoantibodies (MAAs). The presence of MSA such as anti-MDA5, anti-Mi-2, and anti-NXP2 is helpful for the diagnosis of dermatomyositis, but can indicate the clinical phenotype that may occur during disease progression. However, clinical applications are not yet widely used.

## Disease progression and systemic impact

### Muscle necrosis and regeneration cycle

Following the initial immune-mediated vascular injury, the affected muscles undergo repeated *cycle of necrosis and regeneration* [12]. As a result, active muscle fiber necrosis is caused by cytotoxic T cells and inflammatory cytokines such as tumor necrosis factor alpha and interferon gamma (TNF-α, IFN-γ). In response, satellite cells initiate regenerative processes for damaged myocytes, but the persistent inflammatory environment impairs effective repair, leading to repeated injury. Over time, this cycle results in chronic tissue damage, which is characterized by *fibrosis and irreversible muscle atrophy* [12]. Initially, these pathologic changes manifest as symmetric proximal muscle weakness and a decline in functional capacity. In advanced stages, patients begin to struggle with daily activities such as lifting objects or climbing stairs, which highlights the impact of this condition on an individual’s quality of life.

### Calcinosis cutis

A notable complication of dermatomyositis is calcinosis cutis, which involves the abnormal deposition of calcium hydroxyapatite crystals within soft tissues. In juvenile dermatomyositis, calcinosis is associated with delayed immunosuppression, while in adults, it typically manifests in advanced cases of the disease [13]. Calcinosis presents as firm nodules under the skin or deep tumoral deposits in muscle or fascia that can cause ulceration, recurrent infections, and restriction of joints [14]. In pediatric populations, calcinosis can lead to chronic pain and impaired growth, underscoring the importance of *timely immunosuppressive treatment*. These complications increase the morbidity rate of DM significantly, but early intervention can help reduce the risk of calcinosis.

### Interstitial lung disease (ILD)

Systemic involvement can also occur independently of muscle atrophy, making a timely diagnosis less straightforward. One distinct systemic feature is *interstitial lung disease (ILD)*, one of the main factors contributing to a poor prognosis for patients with dermatomyositis [15]. Although presenting subtly in earlier stages with nonspecific symptoms such as dyspnea and cough, it progresses rapidly to acute respiratory failure—especially in patients who have anti-MDA5 antibodies. The risk of developing *rapidly progressive interstitial lung disease* is significantly higher in patients positive for anti-MDA5 antibodies, at 75% compared to just 25% in those who test negative for them [16]. However, early administration of tofacitinib can improve the prognosis of patients with anti-MDA5 antibodies, stressing the importance of prompt diagnosis and intervention.

Pathological findings in ILD include alveolar septal fibrosis, capillaritis, and chronic inflammatory infiltrates, often mimicking microangiopathy seen in skeletal muscle [17]. Therefore, recognizing atypical presentations of ILD and incorporating antibody testing in earlier diagnostic stages can assist in identifying high-risk individuals.

### Cardiac involvement

DM has significant implications on the cardiovascular system, as it induces inflammation of the myocardium, conduction system, and coronary microvasculature. This inflammatory response leads to the development of *arrhythmia and dilated cardiomyopathy* [18]. However, cardiac involvement is often *subclinical*, which contributes to increased mortality in DM. In a study with 160 participants diagnosed with polymyositis and dermatomyositis, it was found that 22% of fatalities were attributed to cardiovascular complications [19]. This makes routine screenings involving electrocardiograms (ECGs) vital, as abnormalities can be detected alongside advanced imaging such as magnetic resonance imaging (MRI) if needed. However, there remains a lack of a standardized protocol for diagnosing myocardial involvement in DM, partly due to the absence of a clear definition for what constitutes cardiovascular involvement.

### Gastrointestinal (GI) complications

The potential for multi-organ involvement goes beyond the lungs and heart, as the gastrointestinal system represents another serious, unrecognized manifestation of DM stemming from *vasculitis of mesenteric circulation* [20]. GI involvement can occur both in children and adults, predominantly in women. In a 30-year retrospective cohort study of 188 DM patients, only three developed severe gastrointestinal complications. The symptom upon presentation was abdominal pain, which subsequently progressed to digestive hemorrhage that required surgery and immunosuppressive treatment. Unfortunately, all three patients succumbed to their conditions, two from GI complications and one from sudden death [20]. However, the small sample size limits generalizability and may not present the same across diverse patient populations. GI involvement is often limited to dysphagia, but severe cases can lead to perforation and ulceration, posing a significant risk to life.

## Pathological and imaging correlates

### Histological findings

Diagnosis of dermatomyositis is heavily reliant on histological imaging, imaging tools, and serologic testing. Collectively, these tools play a vital role in both diagnosis and monitoring of DM, with *muscle biopsy being essential for definitive diagnosis*, especially when serologic testing is inconclusive. Histological findings typically reveal features such as *perifascicular atrophy*, reduced capillary density, and perivascular inflammation. One can also find a large infiltrate presence made up of CD4 + T lymphocytes, B cells, and macrophages that surround muscle fibers, creating the distinguishing feature of DM, as this attributes to perivascular atrophy. Complement activation is seen around intramuscular blood vessels and deposits onto endomysial capillaries, contributing to the characteristic microangiopathy [21]. A correct diagnosis of DM would require a comprehensive immunopathological analysis of muscle biopsies, which can offer insight into the most appropriate therapeutic decision.

### Magnetic resonance imaging

While muscle biopsies remain an important tool for diagnosing dermatomyositis, *magnetic resonance imaging* is now favored for its ability to help identify active inflammation and help guide muscle biopsy site selection. Disease stage strongly influences the appearance of muscle on MRI, with distinct features seen in active versus chronic DM. In earlier phases of DM, T2-weighted and STIR sequences can detect increased edema-like signal intensity resulting from an increase in water content in affected tissues, which signifies ongoing myositis [22]. *Whole-body MRI* can provide a complete assessment of inflammation present in the body and is used to monitor the progression of the disease. It can also detect subclinical disease activity, allowing for early intervention to prevent disease progression. In chronic stages of DM, T1-weighted images show irreversible muscle fatty atrophy and fascial thickening [23]. Nonetheless, there is still a large amount of subjectivity present in interpreting MRI scans, as atrophy and fatty replacement can also signify the natural aging process, further complicating accurate diagnoses in elderly patients.

### Serological testing

Serological testing plays a role in both diagnosis and prognosis, as elevated serum levels of muscle enzymes demonstrate skeletal muscle involvement. Tested enzymes are typically *creatine phosphokinase (CK), aldolase, aspartate transaminase (AST), and alanine transaminase (ALT)*, as they can indicate active myositis. However, it is important to note that elevated serum levels are not specific for myositis, nor do normal muscle enzyme serum levels exclude myositis [24]. Specific DM markers can come from identifying *MSAs and myositis-associated autoantibodies (MAAs)*. The presence of MSAs such as anti-MDA5, anti-Mi-2, and anti-NXP2 can aid in the diagnosis of DM but can indicate clinical phenotypes that may present upon disease progression [10]. Using a combination of these tools—imaging, histology, and serologic testing—can lead to an *early diagnosis of DM*, help clinicians predict complications, and individualize treatment plans. This multifaceted approach is important as patients may present with non-classical DM features or non-specific systemic signs, and early diagnosis can prevent irreversible damage.

### Diagnostic challenges and delays

Early diagnosis of DM is challenging due to its *insidious onset of nonspecific symptoms*, such as fatigue, progressive symmetric proximal muscle weakness, and various characteristic cutaneous findings. The most salient early symptom of dermatomyositis is *muscle weakness*, often developing subacutely [25]. This weakness usually progresses over several weeks to months [25, 26]. Overt manifestations, however, such as muscle atrophy and diminished reflexes, generally manifest in more severe cases, contributing to delays in clinical diagnosis [25].

Concurrently, cutaneous manifestations characteristic of dermatomyositis may appear prior to muscle involvement or alongside it. Such characteristic features, including the *heliotrope rash, periorbital edema, Gottron’s papules, facial erythema involving the nasolabial folds, and the shawl or V-sign*, are often overlooked or misattributed, particularly in patients with darker skin tones where erythema may be more difficult to detect [25, 27, 28]. Additionally, patients may have periungual telangiectasias, diffuse poikiloderma, photosensitivity, and symptoms like the *Holster sign or mechanic’s hands*, all of which are quite particular but must be recognized by dermatologists [25–27].

### Clinical presentation variability

In many cases, *cutaneous findings are the first visible sign of disease*, underscoring the importance of dermatologists in initiating early evaluation [28]. However, the diverse clinical patterns and overlap with other connective tissue diseases can obscure diagnosis. Facial erythema may mimic lupus, and poikiloderma may resemble psoriasis or sun damage, especially in early stages or atypical presentations [28]. In this sense, many clinicians view *nasolabial involvement* as a distinguishing factor to separate dermatomyositis from the malar rash infamously associated with cutaneous lupus erythematosus [28]. This distinction is particularly useful earlier in the disease course, when poikiloderma presents as erythema [28]. Despite these sorts of distinguishing factors, additional symptoms, like Raynaud’s phenomenon, interstitial lung disease, esophageal dysfunction, or even cardiac signs, may converge, complicating the differential diagnosis of dermatomyositis [25].

## Differential diagnosis complexity

### Overlap with other inflammatory myopathies

Diagnosing dermatomyositis can be challenging because of the clinical presentation’s frequent overlap with a variety of immunological, endocrine, and neuromuscular conditions. Other *idiopathic inflammatory myopathies (IIMs)* can exhibit comparable muscular symptoms, but they differ in disease course and pathology [25]. In contrast, dermatomyositis usually features symmetric proximal muscle weakness and characteristic skin rashes. *Clinically amyopathic dermatomyositis* can be challenging to distinguish from other dermatologic or autoimmune disorders, especially in the absence of EMG or biopsy confirmation, as it typically presents with characteristic skin findings but lacks muscle weakness and elevated muscle enzymes [25, 26]. However, in the absence of cutaneous manifestations, rare subtypes such as *"dermatomyositis sine dermatitis"* can present solely with muscular involvement, posing significant diagnostic challenges and often leading to misclassification as polymyositis or other muscular dystrophies [26].

### Systemic disease overlap

Furthermore, extramuscular issues caused by dermatomyositis, such as interstitial lung disease, gastrointestinal dysfunction, or cardiovascular symptoms, might be mirrored by those found in cases of *anti-synthetase syndrome* or lupus [29]. Even rashes like flagellate erythema or periungual erythema can coexist with conditions like scleroderma, Still's disease, or adverse drug or food interactions [28]. Clinicians must maintain a broad differential and use multimodal diagnostics early in the evaluation process due to the clinical diversity across DM subtypes and their overlap with numerous systemic diseases.

### Non-autoimmune myopathies

The fact that dermatomyositis shares overlapping symptoms with other non-autoimmune myopathies that must be excluded in order to confirm the diagnosis further complicates matters. *Inclusion body myositis (IBM)* typically presents with significant muscular atrophy and asymmetric distal muscle weakness [25, 27]. This pattern contrasts with dermatomyositis, which more commonly involves symmetric proximal muscle groups [25, 27]. *Drug-induced myopathies* caused by agents such as statins, glucocorticoids, or antimalarials might result in increased enzymes and muscular weakness, although they usually lack cutaneous manifestations and resolve upon discontinuation of the offending medication [25]. With proximal weakness and elevated enzymes, *endocrinopathies* such as hypothyroidism can resemble dermatomyositis, although measuring thyroid-stimulating hormone (TSH) levels readily distinguishes them [27]. While illnesses like polymyalgia rheumatica cause stiffness and pain without actual muscular weakening or high creatine kinase levels, *neurologic disorders* like myasthenia gravis or motor neuron disease may contribute to weakness without concomitant signs of muscle inflammation or myositis [25].

According to Yang [29], *anti-synthetase syndrome* can strongly resemble dermatomyositis, particularly when characteristics such as ILD, arthritis, Raynaud’s phenomenon, and mechanic's hands predominate. When cutaneous signs occur after muscle weakness or when biopsy and autoantibody testing are neglected or delayed, misdiagnosis is frequently the result. In order to properly diagnose dermatomyositis and start an appropriate treatment, a combination of clinical pattern identification, histology, autoantibody profiling, and exclusion of differential diagnoses is essential.

## Diagnostic complexity and testing requirements

### Evolution of diagnostic and classification criteria in dermatomyositis

Early diagnostic criteria for idiopathic inflammatory myopathies were largely based on the Bohan and Peter criteria. First published in 1975, the Bohan and Peter criteria provided a foundational framework for distinguishing dermatomyositis from other IIMs by integrating characteristic DM skin manifestations and defining categories of definite, probable, and possible diagnoses [31]. They divided IIMs into five groups:

1. Primary idiopathic polymyositis (PM),
2. Primary idiopathic dermatomyositis (DM),
3. DM/PM associated with neoplasia,
4. Childhood DM/PM associated with vasculitis,
5. DM/PM with associated collagen-vascular disease [32, 33]

Core elements when categorizing a diagnosis include symmetric proximal muscle weakness, elevated serum muscle enzymes, electromyographic abnormalities, muscle biopsy evidence of inflammation, and pathognomic skin manifestations in DM [31]. Although groundbreaking at the time, the Bohan and Peter criteria did have their limitations. Particularly in identifying clinically amyopathic dermatomyositis, juvenile subtypes, and patients with minimal muscle involvement.

To improve specificity, the European Neuromuscular Center (ENMC) later proposed new criteria, requiring histological confirmation in conjunction with clinical and laboratory criteria for IIM classification [31]. This was allowed for significant improvement in diagnostic consistency but relied heavily on biopsy expertise.

The most significant advancement came with the 2017 European League Against Rheumatism/American College of Rheumatology (EULAR/ACR) Classification Criteria. A significant improvement over past criteria, allowing for high-probability diagnosis without biopsy [31]. Unlike earlier frameworks, the EULAR/ACR criteria allowed each diagnostic feature to be weighted according to predictive value. The presence of hallmark skin findings increases the probability of a DM classification, allowing for early diagnosis of patients with CADM or minimal muscle involvement.

### Multimodal diagnostic approach

The detailed, *multimodal approach* that incorporates laboratory indicators, imaging, electrophysiology, biopsy, and clinical findings contributes to the diagnostic complexity of dermatomyositis. This comprehensive approach is crucial given the variety of disease subgroups, such as *juvenile dermatomyositis*, *classical dermatomyositis*, and clinically amyopathic forms like CADM, in which patients may exhibit skin symptoms without any discernible muscle weakness [27].

*Clinically amyopathic dermatomyositis* is further classified into amyopathic and hypomyopathic subtypes. Patients with *amyopathic dermatomyositis* show neither clinical symptoms nor laboratory evidence of muscle involvement, whereas those with *hypomyopathic dermatomyositis* lack overt muscle weakness but demonstrate subclinical muscle inflammation on imaging, laboratory tests, and biopsy [25]. Ancillary testing is crucial to the diagnosis in these situations.

### Laboratory testing

Elevated enzyme levels, including but not limited to *creatine kinase (CK), aldolase, lactate dehydrogenase (LDH), aspartate transaminase (AST), and alanine transaminase (ALT)*, reflect underlying muscle inflammation, commonly preceding notable weakness [26]. Further support of autoimmune causative origin includes elevated erythrocyte sedimentation rate (ESR), C-reactive protein (CRP), and positive antinuclear antibody (ANA) [27]. Nevertheless, the integration of additional modalities is necessary for a precise diagnosis because these markers are not specific.

## Diagnostic approaches

### Electromyography (EMG)

*Electromyography (EMG)* is paramount in identifying affected muscles and separating dermatomyositis from other neuropathic disease processes. Abnormalities in EMG common to dermatomyositis comprise spontaneous fibrillations, polyphasic motor unit potentials (short-duration), and positive sharp waves, among others; despite this, *up to 11% of patients may have normal or nonspecific results* [25].

### Magnetic resonance imaging

Another applicable tool is *magnetic resonance imaging*, which provides a noninvasive visualization of inflamed and edematous muscle tissue areas [26].

### Muscle and skin biopsy

*Muscle biopsy remains the diagnostic gold standard* for dermatomyositis, once suspected, usually showing inflammatory perivascular and perimysial infiltrates paired with atrophy of perifascicular muscle areas, and even microangiopathic changes, such as capillary complement deposition [25]. Similarly, *skin biopsies*, utilized in the case of "cutaneous-dominant" cases, may reveal pathologic vacuolar changes within the epidermal-dermal junction as well as mucin deposition, which bears histological resemblance to lupus [25].

### Autoantibody profiling

In addition to histological and imaging studies, *autoantibody profiling* is crucial: myositis-specific autoantibodies such as anti-Mi-2, anti-MDA5, anti-TIF-1γ, and anti-NXP2 not only assist in diagnosis but also predict organ involvement, disease severity, and malignancy risk [26, 28]. For instance, *anti-TIF-1γ* is linked to cancer-associated DM, while *anti-MDA5* is associated with rapidly progressive ILD, particularly in CADM patients [26]. Given their prognostic utility, autoantibody testing is recommended as part of the standard workup, though access to comprehensive panels is limited to specialty labs [8]. These challenges are heightened by variation in access to subspecialists such as rheumatologists, neurologists, dermatologists, and pulmonologists—often essential for diagnosis and disease management [26]. In the absence of tightly integrated, interdisciplinary care, the diagnostic process can remain *fragmented and significantly delayed*.

## Disease rarity and recognition challenges

### Low incidence rates

Another contributing factor to the diagnostic delay and challenges of dermatomyositis is that the disease is still not widely recognized, perhaps due to its *rarity*. For instance, a retrospective analysis conducted in Olmsted County, Minnesota, over 40 years, found an incidence of *9.63 per 1,000,000 individuals* [25]. Low incidence rates like these exacerbate diagnostic ambiguity and complexity, meaning that *most physicians will only see a few cases of dermatomyositis, if any, over their career span*. This rarity makes it difficult to maintain up-to-date diagnostic criteria, and "classic" clinical presentation vignettes will prove difficult [25].

### Research and guideline limitations

Large-scale clinical research and regular updates to guidelines have helped autoimmune disorders like lupus and rheumatoid arthritis, but *idiopathic inflammatory myopathies* like dermatomyositis have not profited from the same advancements [29]. There is still variation in diagnosis and therapy because the development of reliable diagnostic algorithms has been hindered by a lack of scientific and clinical research.

### Subtype complexity

The variety of subgroups within dermatomyositis and the previously described overlapping symptoms, including *amyopathic dermatitis*, *polymyositis*, or *dermatomyositis sine dermatitis*, are further factors contributing to this diagnostic uncertainty. The difficulty, as Yang [29] points out, is not only in diagnosing dermatomyositis but also in ruling out illnesses that resemble it or fall into classification gray areas. Patients who do not exhibit the entire triad of skin involvement, muscle weakness, and laboratory abnormalities are at an *elevated risk for misdiagnosis or delayed diagnosis* due to varying diagnostic criteria across subtypes. In that capacity, both clinical care and research development must prioritize improving diagnostic precision, although this is made more difficult by the disease’s naturally low occurrence. Consequently, despite the possibility of severe systemic involvement and long-term disability, dermatomyositis continues to be a *rare and poorly known disease* [29].

## Clinical inexperience and healthcare system barriers

### Provider education gaps

Clinical inexperience, both among generalists and dermatologists alike, is a major impediment to diagnosing dermatomyositis. As previously mentioned, many clinicians are unfamiliar with dermatomyositis’ nuanced clinical and histological presentations, especially in patients who lack overt muscle weakness and do not fit the classic presentation of dermatomyositis, namely through the absence of characteristic features like *Gottron’s papules* [30]. Even in the case of obvious cutaneous involvement, such patients may be immediately referred to rheumatology instead of being extensively worked up within a dermatological framework, leading to *fragmented care* [30]. Dermatologists may not recognize that *newer DM-specific autoantibodies* now characterize the majority of cases, leading to missed diagnostic opportunities [30]. As such, systemic screening for associated malignancy or interstitial lung disease may be overlooked entirely, despite well-established links between dermatomyositis and these complications [25]. The *underutilization of myositis antibody panels* is another consequence of limited provider education and insufficient availability of commercial tests, which are often constrained by sensitivity and specificity issues [30].

### Healthcare system structure

Systemic obstacles to receiving specialized treatment exacerbate these practice gaps. Diagnostic delays are considerably longer in *gatekeeper-style healthcare systems*, where primary care authorization is required for referrals to specialists [31]. Patients with rare diseases like IIMs frequently exhibit nonspecific symptoms such as muscle soreness or exhaustion that are not initially linked to a significant underlying illness. Consequently, doctors might not associate recurrent vague complaints with an uncommon illness. Patients in gatekeeper systems took longer to receive a diagnosis than those in more open-access healthcare models, according to [34]. This accentuates how the manner in which health systems are set up can have a *direct effect on how quickly rare diseases* like dermatomyositis are diagnosed. Without coordinated access to rheumatologists, neurologists, pulmonologists, and dermatologists—all of whom are crucial to comprehensive care—diagnosis is difficult even once patients are referred [26]. Despite this, there exists a need to bolster clinical vigilance and early cross-specialty evaluation to inhibit underdiagnosis, misdiagnosis, and delayed diagnosis, as highlighted by the disease’s thoroughly described constellation of progressive, erratic, and occasionally deceptive symptoms.

### Access and insurance barriers

Timely diagnosis and treatment of dermatomyositis are often obstructed by systemic healthcare barriers, such as *insurance limitations and delayed access to specialists*. Given dermatomyositis's low incidence, optimal care frequently requires evaluation by a *multidisciplinary team* and, in some cases, referral to tertiary care centers [25], which in turn delays the diagnostic process [25]. However, securing access to subspecialists (e.g., rheumatologists, dermatologists, neurologists, etc.) can prove challenging, especially in regions with limited specialist availability. In many healthcare systems, especially gatekeeper models where primary care providers control referrals, these steps can be delayed. Namsrai [34], found that patients in such systems experience longer diagnostic timelines compared to those in non-gatekeeper models, largely due to restrictions in accessing specialty services. These structural delays can have significant consequences, allowing disease progression before proper interventions are initiated. As Schmidt [26] emphasizes, due to the nature of dermatomyositis as a disease that oftentimes requires diagnostic confirmation via additional testing and personalized care regimens, there must be a push for *coordinated care*.

### Consequences of delayed diagnosis

Delayed diagnosis of musculoskeletal conditions starts a cascade of adverse consequences that extends far beyond the primary pathology, with significant impact on progressive muscle deterioration, systemic organ involvement, soft tissue complications, and significant psychosocial distress [35]. The first and most direct consequence of delayed intervention in musculoskeletal disorders is the sudden onset of *structural and functional muscle damage*. This is characterized by a triad of *fibrosis, muscle atrophy, and consequential weakness*, each of which significantly diminishes physical function [36].

### Structural muscle changes

*Fibrosis* is characterized by the excess accumulation of extracellular matrix proteins; it fundamentally alters the biomechanical properties of muscle tissue [37]. This fibrotic infiltration diminishes the elasticity and contractile efficiency of muscle fibers [38]. The alterations in the architecture of muscle fibers impede the efficiency of the transduction force along the sarcomere, leading to a decline in overall muscle strength [39]. *Muscle atrophy* further exacerbates the functional decline due to the reduction in muscle fiber size and number [40]. With regard to consequential weakness, a common manifestation is *joint contracture*, which is a condition where the normal movement of a joint is restricted due to shortening or changes in the soft tissues [41]. This includes alterations in muscle, ligaments, and tendons. Joint contractures are a common consequence of delayed diagnosis, significantly limiting the range of motion and impacting daily activities [42]. As a result, individuals can develop capsular fibrosis, muscle stiffness, and atrophy. The mechanism of contracture formation involves both collagen cross-linking due to fibrosis and tissue shortening because of muscle atrophy [43].

### Serum markers of muscle damage

Serum markers such as *creatine kinase (CK) and aldolase* are typically elevated in patients with delayed diagnosis, reflecting ongoing muscle injury [44]. Creatine kinase is crucial for energy production in muscle cells during contraction. Elevated levels of CK in the blood can indicate muscle damage, which is common in patients with delayed diagnosis of musculoskeletal conditions with ongoing muscle cell damage [26]. Aldolase is a glycolytic enzyme present in muscle tissue, assisting in the metabolic pathway for breaking down glucose for energy. Similar to CK, elevated aldolase levels can indicate muscle cell damage.

## Musculoskeletal complications

### Joint contractures

A *joint contracture* is a condition where the normal movement of a joint is restricted due to shortening or changes in the soft tissues [41]. This includes alterations in muscle, ligaments, and tendons. The mechanism of contracture formation involves both collagen cross-linking due to fibrosis and tissue shortening as a result of muscle atrophy. *Capsular fibrosis* involves the thickening and scarring of the joint capsule. This is a result of chronic inflammation and excessive deposition of collagen and other extracellular matrix components, resulting in reduced joint space and mobility [45]. In addition to capsular fibrosis, *collagen cross-linking* increases the stiffness and reduces the elasticity of the tissues. This is a result of collagen fibers in the muscles, tendons, and ligaments forming abnormal cross-links [46].

This makes it harder to stretch or move the affected joint, leading to further limitations in range of motion. Some possible interventions for addressing contractures and related issues include:

- Physical therapy: The primary intervention to improve range of motion, reduce stiffness, and strengthen weakened muscles. Treatment involves a combination of strengthening exercises and manual therapy techniques to address capsular fibrosis.
- Splinting: Used to support and maintain joint position.
- Surgical release: In severe cases where physical therapy is insufficient, surgical intervention may be necessary. This involves the surgical cutting or release of the contracted tissues to restore greater range of motion [47].

Surgical excision may also be necessary for the removal of painful calcinosis cutis as well as to prevent future infections.

### Calcinosis cutis

Beyond pain, these deposits create an environment of *localized inflammation* and *increased susceptibility to secondary infections* due to bacterial colonization [48]. The loss of skin integrity facilitates microbial invasion, leading to recurrent infections. While *surgical intervention* may not guarantee a permanent resolution and carries procedural risks, excision of calcified nodules may be necessary in severe cases to relieve pain, prevent infection, and restore function.

*Chronic inflammation* in dermatomyositis contributes not only to local musculoskeletal damage but also to *systemic complications*. The interplay between persistent immune activity and tissue injury reinforces the importance of *early diagnosis and intervention* to prevent these downstream effects.

## Systemic comorbidities in delayed diagnosis

Delayed diagnosis of dermatomyositis increases the risk and severity of *multisystem involvement*, including the pulmonary, cardiac, and malignant complications [49].

### Myocarditis

Myocardial inflammation may lead to *arrhythmias*, *conduction abnormalities*, and *heart failure* [50]. Diagnostic modalities include ECG, troponin levels, cardiac MRI, and BNP levels. *Undiagnosed myocarditis* remains a silent yet serious contributor to morbidity in dermatomyositis patients.

### Malignancy risk

Dermatomyositis is strongly associated with malignancy, especially in adults. *Chronic inflammation and immune activation* foster an environment conducive to oncogenesis. Increased levels of cytokines, arachidonic acid metabolites, chemokines, and *reactive oxygen species* may damage DNA and promote *genetic mutations* linked to cancer [51–53].

### Psychological and functional burden

The effects of delayed diagnosis extend beyond physical illness to cause *substantial psychological distress* and *reduced quality of life*. *Persistent fatigue*, a common and debilitating symptom, disrupts cognitive and physical function [54]. This symptom often stems from chronic inflammation and musculoskeletal degeneration. Additionally, *visible skin lesions*, *muscle atrophy*, and *joint deformities* can lead to disfigurement, fostering *low self-esteem, social withdrawal, and shame* [55].

Functional limitations from *joint stiffness*, *pain*, and *muscle weakness* interfere with daily activities. Many patients experience a *loss of independence*, which contributes to *psychological decline* and *reliance on caregivers* [56].

Chronic illness increases the risk of *depression and anxiety*, which may exacerbate physical symptoms through *stress-induced disease flares* [57, 58]. Routine use of validated tools such as the *PHQ-9* and *GAD-7* can help identify psychological comorbidities before they escalate.

## Immunosuppressive therapies and multidisciplinary management

### Pharmacologic treatment options

*Immunosuppressive therapy* remains the cornerstone of dermatomyositis management. First-line treatment typically includes *corticosteroids* (e.g., prednisone), which provide rapid anti-inflammatory effects [59]. However, due to long-term side effects, *steroid-sparing agents* like *methotrexate* and *azathioprine* are frequently used in combination therapy [60].

*Intravenous immunoglobulin (IVIG)* has shown efficacy in steroid-refractory cases, as supported by randomized controlled trials [61]. More recent biologic therapies, such as *rituximab* (anti-CD20), have demonstrated benefit in specific subgroups, including those with *anti-Jo-1 myositis* [62]. Conversely, *abatacept* showed early promise but failed to meet clinical endpoints in trials [63]. Retrospective studies show that *early treatment* is associated with greater one-year remission rates, faster symptom resolution, and reduced relapse risk [64].

### Physical and occupational therapy

Adjunctive *physical and occupational therapy (PT/OT)* plays a vital role in preserving muscle strength and mobility. Studies demonstrate that early PT improves function and does not exacerbate inflammation [65, 66]. Regardless of disease stage, PT focused on *range of motion, strength training, and conditioning* has shown consistent benefits. Given dermatomyositis’s *multisystem involvement*, management should involve a *multidisciplinary care team*, including specialists in *rheumatology, dermatology, neurology, pulmonology, surgery*, and *rehabilitation medicine* [67]. Early integration of specialty care improves outcomes by allowing for targeted interventions, *early palliative strategies*, and *coordinated monitoring* [68].

## Conclusion

Early diagnosis, timely initiation of immunosuppressive therapy, and coordinated interdisciplinary care are central to improving outcomes in dermatomyositis. A high index of suspicion is necessary, especially in patients with non-specific symptoms or isolated skin findings. Because *25% of patients* may present with malignancy [69], screening and early antibody testing are essential. Clinicians must be trained to recognize subtle cutaneous signs, especially in *darker skin tones,* to prevent diagnostic delays [70, 71].

Collaboration between dermatologists, rheumatologists, neurologists, and pulmonologists is vital for comprehensive care and ensures timely intervention [72]. Implementation of structured screening protocols and multidisciplinary order sets at diagnosis can streamline treatment. Further research is needed to expand our understanding of dermatomyositis pathogenesis and to develop more precise *biomarkers* for early detection [73]. Through *early intervention*, *targeted therapy*, and *team-based care*, we can significantly reduce long-term complications and improve the quality of life for individuals affected by this debilitating disease.

## Data Availability

Not applicable.
